# Adjunctive use of a collagen membrane for surgical closure of oronasal fistulae in dogs with challenging local conditions: a case series

**DOI:** 10.3389/fvets.2026.1854434

**Published:** 2026-06-23

**Authors:** Kimiyoshi Okano

**Affiliations:** Yokohama Animal Dental Clinic, Kanagawa, Japan

**Keywords:** canine teeth, collagen membrane, dog, oronasal fistula, periodontal disease

## Abstract

This case series describes the clinical outcomes of adjunctive use of a collagen membrane in the surgical closure of oronasal fistulae (ONF) in dogs presenting with clinical conditions that may compromise stable closure using a standard single-flap technique. Six dogs (eight maxillary canine teeth) with ONF were included in this consecutive clinical case series, selected based on the presence of multiple challenging clinical conditions, often in combination, including marked loss of buccal gingiva, insufficient alveolar bone support, and reduced soft tissue mobility associated with scarring. Following extraction of the affected teeth, a mucoperiosteal flap with adequate tension release was created. A trimmed collagen membrane was then placed on the nasal surface of the mucoperiosteal flap to support the suture line, and primary closure was achieved using a single-flap technique. In all cases, clinical signs associated with ONF, including epistaxis, nasal discharge, and sneezing, resolved within 14–21 days postoperatively. During the available follow-up period, no clinical or oral findings suggestive of fistula recurrence were observed. Although this technique is not intended to replace established closure methods, the adjunctive placement of a collagen membrane on the nasal surface of the mucoperiosteal flap to support the suture line may serve as a temporary supportive measure to stabilize the early healing environment at the suture line during the immediate postoperative period. The collagen membrane does not reduce the need for appropriate flap design or adequate tension release in selected ONF cases with challenging local conditions.

## 1 Introduction

Periodontal disease–associated oronasal fistulae (ONF) involving the maxillary canine teeth are relatively common conditions in dogs (1). In recent years, attempts to preserve affected teeth using periodontal regenerative therapies have been reported; Watanabe et al. (2) however, in routine clinical practice, extraction of the affected tooth followed by primary closure with a mucoperiosteal flap remains the standard treatment approach (3–5).

Although favorable healing outcomes can be achieved with a single-flap technique in many cases, closure failure is more likely under certain clinical conditions. These include situations in which the soft tissues are fragile, the buccal gingiva is minimal or absent and only oral mucosa remains, or the supporting alveolar bone is insufficient, as well as cases requiring revision surgery. Under such circumstances, residual tension at the suture line, together with postoperative mechanical stresses—such as intranasal pressure, sneezing, tongue movement, and mastication—may compromise wound stability and increase the risk of wound dehiscence and recurrence (1, 3, 6, 7).

In cases where multiple local factors coexist, achieving stable closure may be more technically challenging. In such situations, several alternative closure strategies have been described. Soft tissue–based techniques include double-flap procedures and extensive mucoperiosteal flap designs (8). Additionally, methods aimed at reinforcing the barrier between the oral and nasal cavities, such as auricular cartilage grafting, have been reported (7, 9). More recently, adjunctive use of artificial membranes for the repair of acquired periodontal disease–related ONF has also been described; however, consensus regarding their clinical effectiveness has not been established (10).

While these techniques have demonstrated acceptable clinical outcomes, they may require extensive soft tissue dissection or harvesting of graft materials depending on case conditions and surgeon experience, potentially resulting in increased surgical invasiveness and longer operative times. Furthermore, a relatively high degree of operator dependence has been noted, raising concerns regarding procedural reproducibility (7–10).

In this context, the present report focuses on an adjunctive strategy using a collagen membrane as an artificial material placed beneath the suture line to temporarily stabilize the healing environment, while avoiding additional donor-site morbidity and allowing for relatively rapid placement. Importantly, this approach does not reduce the need for appropriate flap design or tension release, but rather serves as an adjunctive measure. This approach was hypothesized to support early postoperative healing, particularly in cases with compromised local conditions or where minor suture line separation might occur. In this case series, this technique was applied to six dogs (eight teeth) with oronasal fistulae, and the clinical course of these cases is described.

## 2 Materials and methods

This study was designed as a retrospective descriptive case series of client-owned dogs diagnosed with oronasal fistulae and treated using a standardized surgical approach.

This case series included eight maxillary canine oronasal fistula sites in six dogs in which closure using a conventional single-flap technique was expected to be technically challenging due to local clinical conditions. Four dogs had unilateral ONFs, whereas two dogs had bilateral ONFs. One unilateral case presented with an apparent ONF associated with a missing maxillary canine tooth, whereas the remaining seven ONF sites were classified as inapparent ONFs associated with retained teeth. The breeds included three Toy Poodles, two Miniature Dachshunds, and one Italian Greyhound.

**Figure 1:**
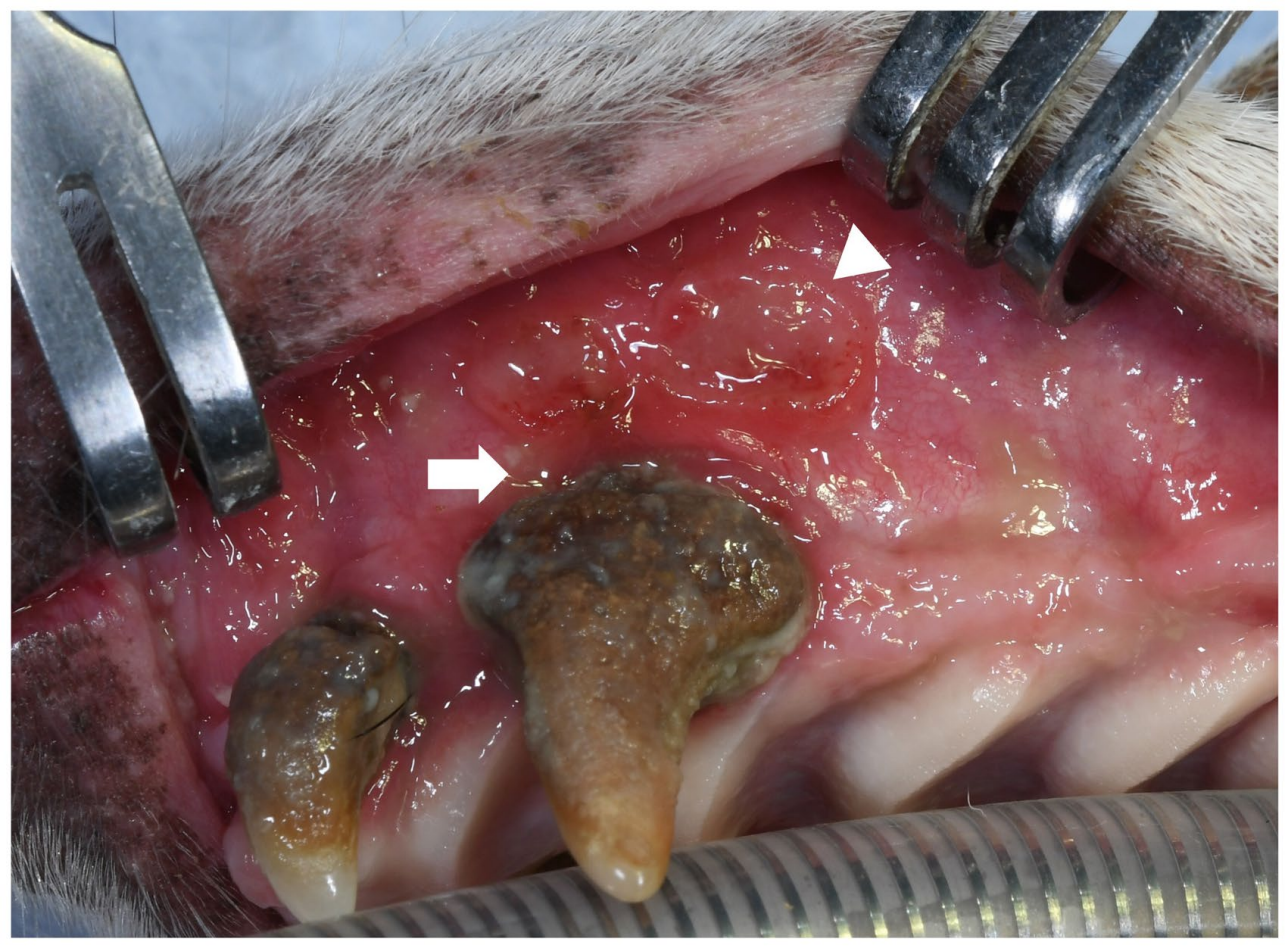
Representative clinical appearance of an oronasal fistula presenting with challenging local conditions (Case 6). The arrow indicates an area where the gingiva has been lost and replaced by oral mucosa. The arrowhead indicates erythematous and ulcerated oral mucosa, which bled easily upon minimal manipulation. In this study, cases presenting with these findings in combination with poor alveolar bone support were included as cases with challenging local conditions.

Cases were selected based on the presence of multiple challenging local conditions, including a large gingival defect, poor alveolar bone support, or reduced soft tissue mobility due to scarring of the gingiva or oral mucosa (Figure 1). These criteria were derived from commonly recognized clinical factors associated with oronasal fistula closure difficulty described in the literature, and no quantitative scoring system was applied. The aim of this case series was not to establish a new classification, but to describe clinical outcomes in cases presenting with these challenging local conditions.

All dogs exhibited clinical signs suggestive of oronasal fistula based on owner history, including epistaxis, nasal discharge, and sneezing. In one Toy Poodle, the maxillary canine tooth had exfoliated spontaneously, allowing direct visualization of the nasal cavity from the oral aspect. Bilateral oronasal fistulas were observed in one Italian Greyhound and one Miniature Dachshund. The dogs ranged in age from 8 to 15 years and weighed between 3.2 and 7.5 kg (Table 1).

**Table 1:** Clinical characteristics and outcomes of dogs with oronasal fistulae presenting with challenging local conditions.

Case No.	1	2	3	4	5	6
Breed	Toy Poodle	Toy Poodle	Toy Poodle	Miniature Dachshund	Miniature Dachshund	Italian Greyhound
Age (years)	8	14	12	12	15	15
Body weight (kg)	3.2	4.4	3.8	5.4	5.2	7.5
Sex	FS	MC	MC	MC	FS	FS
Affected side	Left	Right	Left	Right	Bilateral	Bilateral
Canine status	Present	Missing	Present	Present	Present	Present
Follow-up period	2 months	21 days	4 months	21 days	7 months	6 months

This table summarizes the signalment, affected sites, and clinical outcomes of dogs diagnosed with oronasal fistulae (ONF). Cases were selected based on the presence of clinical conditions that may complicate stable closure using a standard single-flap technique, including large gingival defects, insufficient alveolar bone support, and/or reduced soft tissue mobility associated with scarring. All cases were followed postoperatively, and resolution of clinical signs without recurrence was confirmed during the follow-up period. All cases were evaluated at 14–21 days postoperatively. Additional follow-up beyond this period was available in some cases but not in all.

FS, female spayed; MC, male castrated; ONF, oronasal fistula.

All diagnostic and surgical procedures were performed under general anesthesia.

Dental radiography and periodontal probing were performed in all cases. In dogs with retained canine teeth, oronasal fistula was diagnosed when probing from the palatal aspect resulted in bleeding from the nasal cavity, or when leakage into the nasal cavity was confirmed using a saline irrigation test, in which saline was gently injected through the suspected fistula site and leakage from the external naris was observed (11).

**Figure 2:**
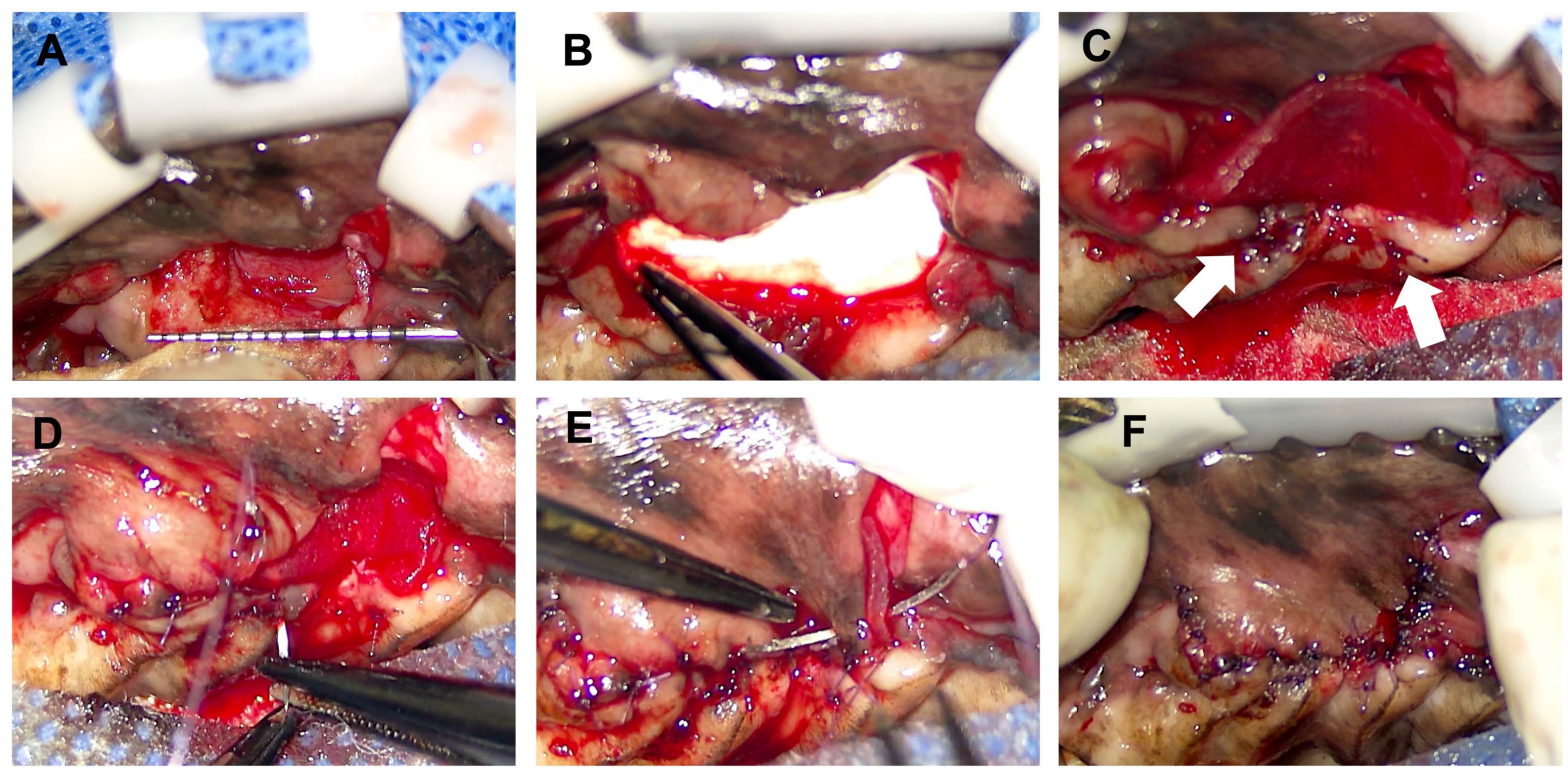
Intraoperative photographs from Case 1 illustrating the adjunctive use of a collagen membrane during closure of an oronasal fistula presenting with challenging local conditions: **(A)** measurement of the recipient site using a periodontal probe to determine the appropriate membrane size; **(B)** placement of the trimmed collagen membrane beneath the suture line, with visible blood absorption; **(C)** fixation of the membrane to the palatal mucosa using simple interrupted sutures placed at the mesial and distal aspects; **(D)** suturing of the horizontally incised flap to the palatal mucosa while incorporating the membrane within the suture line; **(E)** closure of the vertically incised flap, again incorporating the membrane into the sutures; and **(F)** final appearance after closure, confirming the absence of excessive flap tension and residual defects.

Surgical repair of the oronasal fistula was performed following a standard technique involving the creation of a mucoperiosteal flap with vertical releasing incisions at the mesial and distal aspects. In all cases, the vertical releasing incisions extended beyond the mucogingival junction, and periosteal releasing incisions were performed to achieve adequate flap mobility and tension-free closure, although the extent of the incisions varied depending on the local anatomy and defect configuration. Prior to flap closure, the soft tissue margins surrounding the fistula were conservatively debrided and refreshed to remove chronically inflamed or epithelialized tissue and promote tissue apposition. A collagen membrane (Collprotect® membrane, botiss biomaterials GmbH, Zossen, Germany) was not sized according to the dimensions of the osseous defect, as it was not intended to completely cover the defect or function as a guided tissue regeneration barrier. Instead, the membrane was trimmed chairside using a periodontal probe as a reference, based on the length of the transverse incision and intended suture line support area. The membrane was positioned on the nasal surface of the mucoperiosteal flap and extended approximately 1–2 mm beneath the adjacent gingival and bony margins directly beneath the horizontal simple interrupted suture as an adjunctive support. The membrane was stabilized using simple interrupted sutures with 5–0 monofilament absorbable glyconate suture material (Monosyn, B. Braun Aesculap, Tokyo, Japan), incorporating the palatal tissues into the closure. Primary closure was then achieved using the mucoperiosteal flap, and the absence of excessive tension at the suture line was confirmed at the completion of suturing, with closure primarily dependent on appropriate flap design and tension release (Figure 2).

Postoperatively, dogs were fed a soft diet for two weeks, and behaviors that could place mechanical stress on the canine region, such as grasping or holding objects, were restricted.

## 3 Results

All cases were evaluated at 14–21 days postoperatively. Clinical signs including epistaxis, nasal discharge, and sneezing had resolved in all cases at this time point.

Follow-up duration varied among cases. Cases 1, 3, 5, and 6 underwent additional follow-up examinations several months after surgery, whereas the remaining cases were evaluated only during the early postoperative period.

**Figure 3:**
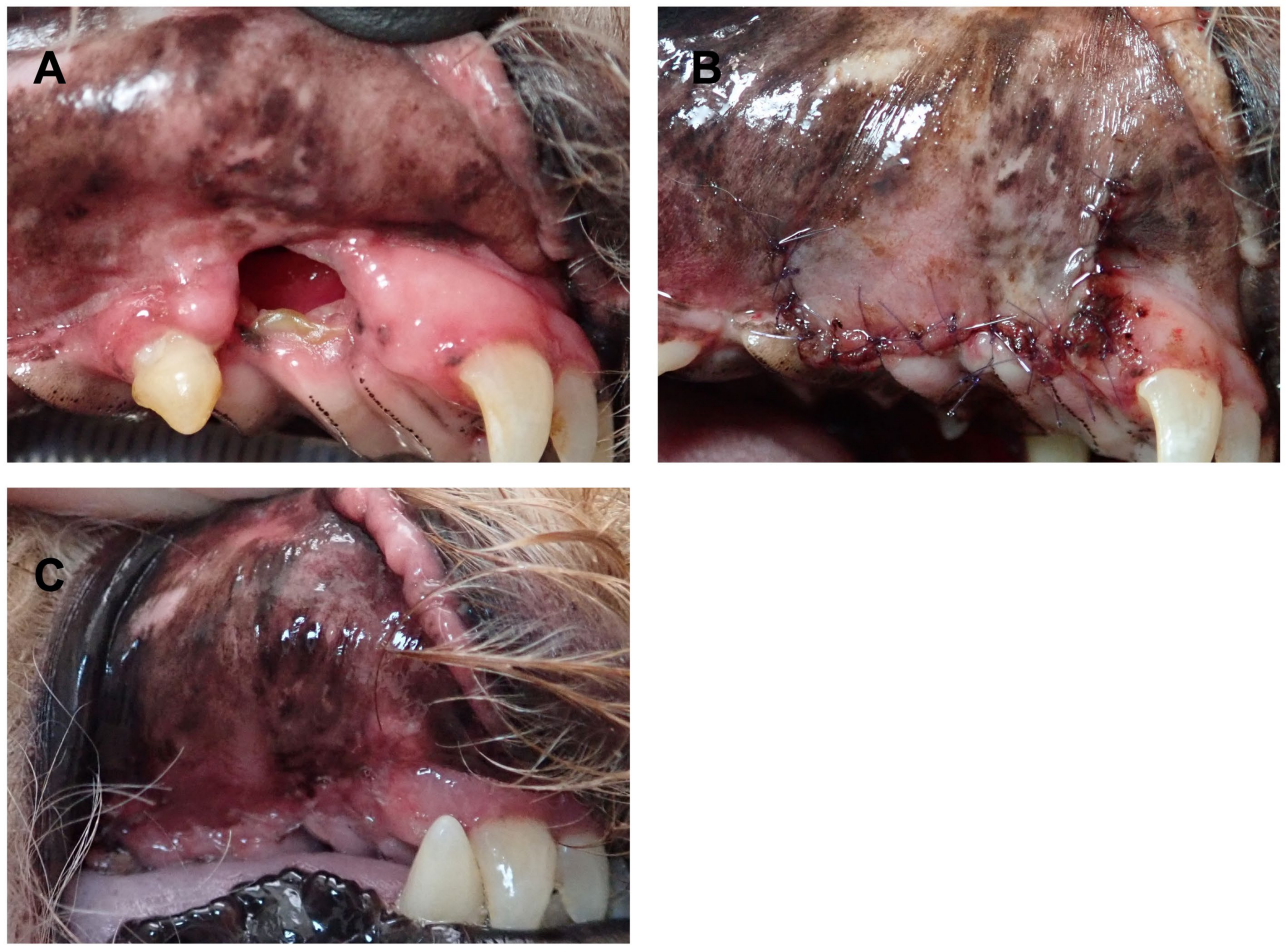
Representative clinical course following adjunctive collagen membrane placement: **(A)** preoperative appearance from Case 2 showing an oronasal fistula; **(B)** immediate postoperative appearance following primary closure using a mucoperiosteal flap; **(C)** 21 days postoperatively demonstrating resolution of clinical signs and maintained mucosal coverage.

No clinical or intraoral findings suggestive of recurrence of oronasal fistula were observed in any case during the available follow-up period (Figure 3).

## 4 Discussion

In this case series, resolution of clinical signs was observed at 14–21 days, and no evidence of recurrence was identified during the follow-up period. The collagen membrane used in this study was placed directly beneath the suture line, and this placement may have temporarily stabilized the local healing environment surrounding the sutured tissues during the early phase of wound healing, as an adjunctive support rather than a primary means of closure, with closure fundamentally dependent on appropriate flap design and tension release.

Shannon (2022) reported a case series evaluating the use of a flexible bone membrane for acquired oronasal fistulas associated with periodontal disease and found that membrane application did not clearly improve surgical success rates. However, the author also noted that multiple confounding factors—including surgeon experience, flap design, methods of membrane fixation, heterogeneity in evaluation time points, and concurrent disease—may have influenced the outcomes, and therefore did not conclude that membrane use itself was ineffective (10). These observations suggest that the clinical role of membranes in oronasal fistula repair may depend not only on the material used, but also on how and in what context the membrane is applied, particularly when used as an adjunctive support beneath the suture line. A key feature of the technique described in the present report is the conceptual positioning of the membrane not as a primary means of fistula closure, but as an adjunctive material intended to support the healing environment at the suture line. In this approach, closure of the fistula relies fundamentally on primary closure using a mucoperiosteal flap, while the membrane is placed directly beneath the suture line with the intention of limiting the progression of early suture-line stress concentration or minor separation into clinically relevant wound failure during the immediate postoperative period.

The collagen membrane used in this study is a porcine dermis-derived collagen matrix characterized by a relatively soft and pliable structure, which facilitates adaptation to irregular defect surfaces and allows for easy incorporation into the suture line. In the present cases, the membrane could be readily positioned beneath the suture line and stabilized without additional fixation devices, suggesting practical advantages in terms of handling and intraoperative workflow. As a xenogeneic biomaterial, collagen membranes may theoretically induce localized inflammatory or foreign body reactions in some cases, although clinically significant adverse reactions appear to be uncommon in both veterinary and human oral surgical applications. Excessive inflammatory responses could potentially compromise local wound healing and contribute to surgical failure (12). In addition, the membrane demonstrated sufficient mechanical strength to withstand traction after suture placement without tearing, and its ability to absorb blood and expand upon contact with fluids may have contributed to local stabilization at the surgical site. These characteristics are consistent with previous reports describing the physicochemical properties, cellular compatibility, and degradation behavior of collagen membranes, including Collprotect®, which have demonstrated favorable cell viability, structural integrity, and gradual resorption profiles (13, 14). Furthermore, collagen membranes of dermal origin have been reported to support vascularization and early tissue integration, which may contribute to stabilization of the local healing environment (15). According to the manufacturer, Collprotect® is classified as an intermediate degradation collagen membrane with an estimated resorption period of approximately 4–8 weeks under standard conditions. However, actual degradation behavior in the oral environment may differ substantially depending on local enzymatic and inflammatory conditions. Recent *in vitro* studies demonstrated that Collprotect® underwent marked structural degradation within approximately 7 days in phosphate-buffered saline and complete degradation within 48 h under bacterial collagenase conditions. Therefore, in the present study, the membrane was intended primarily to support stabilization of the suture line during the early postoperative healing phase rather than to provide prolonged structural support. In contrast, some collagen membranes may exhibit either insufficient mechanical integrity during suturing or resistance to needle penetration, which can complicate intraoperative handling. In the present cases, 5–0 monofilament absorbable glyconate suture material was selected because of its handling characteristics and relatively low tissue reactivity. However, this material is also known to undergo progressive reduction in tensile strength during the postoperative healing period. In challenging ONF cases with compromised local tissue conditions, early stabilization of the suture line may therefore be particularly important during the initial healing phase.

Nevertheless, the most critical factor for successful oronasal fistula repair remains appropriate flap design with adequate tension release, and the use of a collagen membrane does not replace this requirement. When sufficient tension release is not achieved, the addition of a membrane alone may not adequately reduce the risk of suture line dehiscence. Accordingly, the technique described here should be regarded as an adjunctive strategy that supports the initial healing phase rather than replacing standard closure techniques, only after standard principles of oronasal fistula closure have been properly applied. In addition, careful postoperative occlusal assessment is important to ensure that the mandibular canine teeth do not contact the repair site. This consideration may be particularly relevant when adjunctive materials are placed beneath the flap, as subtle changes in vestibular contour or flap thickness may increase the risk of mechanical irritation and wound dehiscence.

Even in cases presenting with challenging local conditions, successful healing using a conventional single-flap technique alone cannot be excluded. However, in cases characterized by extensive gingival defects, insufficient alveolar bone support, or reduced flap mobility due to scarring, achieving stable closure may be more technically challenging. In such situations, the present technique may be selected as a supportive option aimed at minimizing the risk of dehiscence, rather than as a routine modification applied to all oronasal fistula repairs.

Previously reported adjunctive closure strategies for challenging oronasal fistulas include double-flap techniques (5) and the use of auricular cartilage grafts as a physical barrier between the oral and nasal cavities (7, 9). Although these approaches have demonstrated acceptable clinical outcomes, they may involve extensive soft tissue dissection, additional donor-site morbidity, and increased surgical time. In addition, their success can be highly dependent on surgeon experience and case-specific anatomical conditions (7–10). Compared with previously reported adjunctive closure techniques, such as double-flap procedures or auricular cartilage grafting, the present method did not require additional flap extension beyond standard closure or harvesting of donor-site grafts and could be incorporated into standard flap-based closure procedures.

Several limitations of this report should be acknowledged. Case selection was based on clinical and intraoperative findings rather than objective quantitative measurements. Although this reflects real-world surgical decision-making, the absence of a standardized scoring system may limit reproducibility and should be considered when interpreting the results. In addition, quantitative measurements of defect size were not consistently recorded in all cases, which limited objective comparison among lesions. Furthermore, advanced three-dimensional imaging such as computed tomography or cone-beam computed tomography was not consistently performed in this retrospective case series, limiting detailed assessment of osseous defect configuration and postoperative structural changes. This study represents a small case series without a control group, and therefore definitive conclusions regarding the effectiveness of the technique cannot be drawn. Accordingly, the findings should be interpreted as exploratory observations.

Not all cases were followed for the same duration, and follow-up periods varied among sites, with some cases evaluated only during the early postoperative period. Accordingly, the absence of clinical recurrence in cases with shorter follow-up should be interpreted with caution.

The cases included in this study were limited to those presenting with challenging local conditions, and it remains unclear whether this technique is applicable to all forms of oronasal fistula. No direct comparison was made with cases that healed successfully using a standard single-flap technique alone, and further investigation is required to clarify the appropriate indications for this approach.

The technique assumes adequate flap design with sufficient tension release and appropriate suturing, and the influence of surgeon experience and technical proficiency cannot be completely excluded.

Postoperative evaluation was based primarily on clinical signs and gross intraoral findings, and small residual or subclinical communications cannot be entirely ruled out.

Furthermore, the exact *in vivo* persistence and behavior of the collagen membrane used in this study remain unknown, and early resorption or displacement after placement cannot be excluded. As histologic evaluation was not conducted, the extent to which the membrane directly contributed to tissue integration or wound healing cannot be determined. Therefore, this report does not conclude that the collagen membrane directly promoted healing, but rather presents clinical observations suggesting that it may have functioned as a temporary adjunct supporting the early healing environment at the suture line, with closure fundamentally dependent on appropriate flap design and tension release.

Considering these limitations, the present case series is valuable in demonstrating that adjunctive placement of a collagen membrane beneath the suture line is clinically feasible in cases presenting with challenging local conditions and was not associated with clinical recurrence during short- to mid-term follow-up. This technique is not intended to replace conventional oronasal fistula closure methods, but may be considered as one option to support the early healing phase in selected cases with challenging local conditions. Further studies involving larger case numbers and comparative designs are warranted to better define the indications and potential utility of this approach.

## 5 Conclusion

In this case series, surgical repair of oronasal fistulas in dogs presenting with challenging local conditions was performed using a standard single-flap technique with adjunctive placement of a collagen membrane. Resolution of clinical signs was observed in all evaluated cases, and no clinical evidence suggestive of recurrence was identified during the follow-up period. This technique is not intended to replace established closure methods but should be regarded as an adjunctive approach that supports the early healing environment at the suture line when appropriate flap design and sufficient tension release are achieved. In selected cases with challenging local conditions, this method may represent one possible option to consider for supporting the early healing phase and potentially minimizing the risk of wound dehiscence.

## Data Availability

The original contributions presented in the study are included in the article/supplementary material, further inquiries can be directed to the corresponding author/s.
